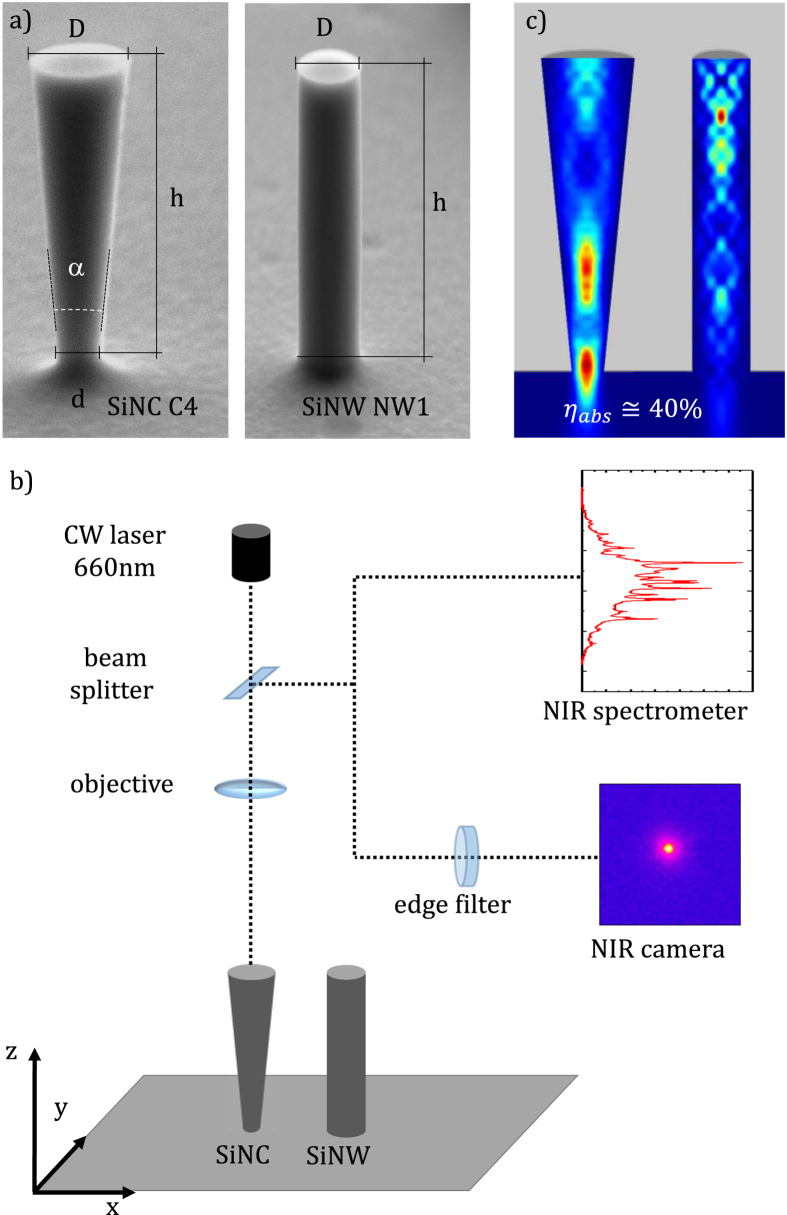# Corrigendum: Observation of strongly enhanced photoluminescence from inverted cone-shaped silicon nanostructures

**DOI:** 10.1038/srep22178

**Published:** 2016-03-10

**Authors:** Sebastian W. Schmitt, George Sarau, Silke Christiansen

Scientific Reports 5: Article number: 17089; 10.1038/srep17089published online: 11262015; updated: 03102016

The original version of this Article contained an error in the title of the paper, where the word “nanostructures” was incorrectly given as “nanostuctures”.

In addition, there was an error in Figure 1. In Figure 1b, the SiNC was partially obscured. The correct Fig. 1 appears below.

These errors have now been corrected in the PDF and HTML versions of the Article.

## Figures and Tables

**Figure 1:**