# Supplementary material for: Observation of strongly enhanced photoluminescence from inverted cone-shaped silicon nanostuctures
Source: Sci Rep. 2015 Nov 26;5:17089. doi: 10.1038/srep17089 (PMC4660596; doi:10.1038/srep17089)
Supplement: Supplementary Information [file srep17089-s1.pdf]

Observation of strongly enhanced photoluminescence from inverted cone-shaped silicon nanostructures

Sebastian W. Schmitt*, George Sarau, Silke Christiansen

Max Planck Institute for the Science of Light, Photonic Nanostructures, Günther-Scharowsky-Str. 1, 91058 Erlangen / Germany

Helmholtz-Zentrum Berlin für Materialien und Energie, Institute of Nano-architectures for Energy Conversion, Hahn-Meitner-Platz 1, 14109 Berlin / Germany

*Corresponding author: sebastian.schmitt@mpl.mpg.de, sebastian.schmitt@helmholtz-berlin.de

Supplementary information S1: SEM micrographs of four SiNCs (tilt 70°) and one SiNW (tilt 45°) fabricated by cryogenic RIE with SF_6 and O_2 chemistry (scale bar is 1µm). Masking is performed with silica spheres (diameter 1µm). Different shapes can be realized by the adjustment of O_2 concentration in the plasma that results in a different amount of under etching. The table gives the geometrical parameters of the produced nanostructures as determined by SEM and an image processing software.

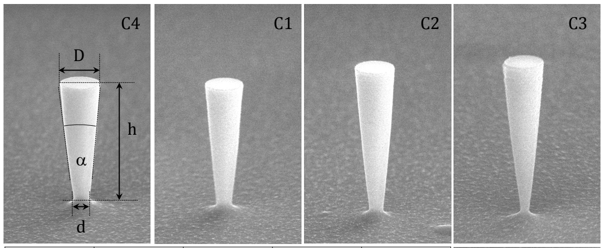

no.	h	D	d	α
	[nm]	[nm]	[nm]	[°]
C4	2928	941	363	5.6
C1	2971	855	317	5.2
C2	3514	914	287	5.1
C3	3567	848	129	5.8
NW1	2918	572	572	0.0

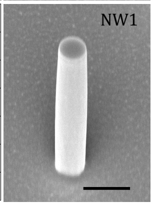

Supplementary information S2: The PL intensity I from C1, C2, C3, and C4 was baseline corrected between 1000 and 1200nm (black line) and fitted (red line) by a sum of multiple Voigt peak profiles where each profile is given by

$$I(\lambda) = \int G(\tau)L(\lambda - \tau)\,d\tau \qquad (1)$$

with

$$G(\lambda) = \frac{\frac{e^{-\lambda^2}}{2\sigma^2}}{\sigma\sqrt{2\pi}} \qquad (2)$$

and

$$L(\lambda) = \frac{\gamma}{\pi(\lambda^2 - \gamma^2)} \qquad (3)$$

Here, G and L are a Gaussian and Lorentzian distribution with the widths σ and γ, respectively. The spectral peak width $\Delta\lambda$ is then given by the approximation $\Delta\lambda = 1.1\gamma + \sqrt{0.9\gamma^2 + 8\ln 2\sigma^2}$[1]. Accordingly the Q-factors were calculated as

$$Q = \frac{\lambda_o}{\Delta\lambda} \qquad (4)$$

where λ_o is the spectral position of the peak maximum.

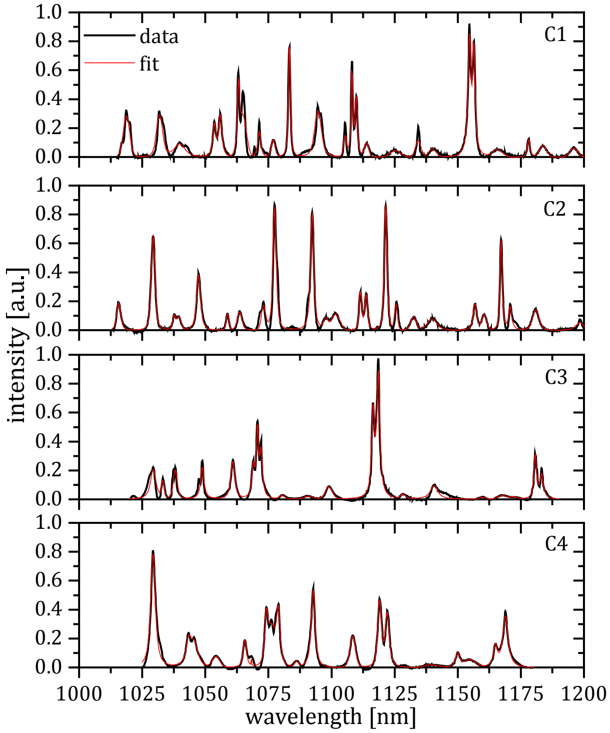

Supplementary information S3: For the mode analysis, broadband dipole pulses (850-1250nm) polarized in x- and z- direction (E_x, E_z) were excited in the maxima of the pump laser absorption in a SiNC with the geometry of C4 and a SiNW with geometry NW1 (compare Fig. 1c). In a and b it can be seen that within the SiNW attached to the wafer, the pulse energy (proportional to E^2) decays much faster i.e. it is by far more optically 'leaky' than the SiNC, which is able to retain more optical energy over a longer time span. c and d show a Fourier transformation of the optical power emitted through the top facet of the SiNW and SiNC for t>600fs. While the spectra for the SiNC for different excitation still shows a strong emission in a multitude of sharp peaks, the emission of the SiNW is much weaker and only a few shallow peaks are visible in the spectrum. This is in good agreement to the PL spectra in Fig. 2a where in contrast to the SiNW the emission of the SiNC shows strong additional peaks.

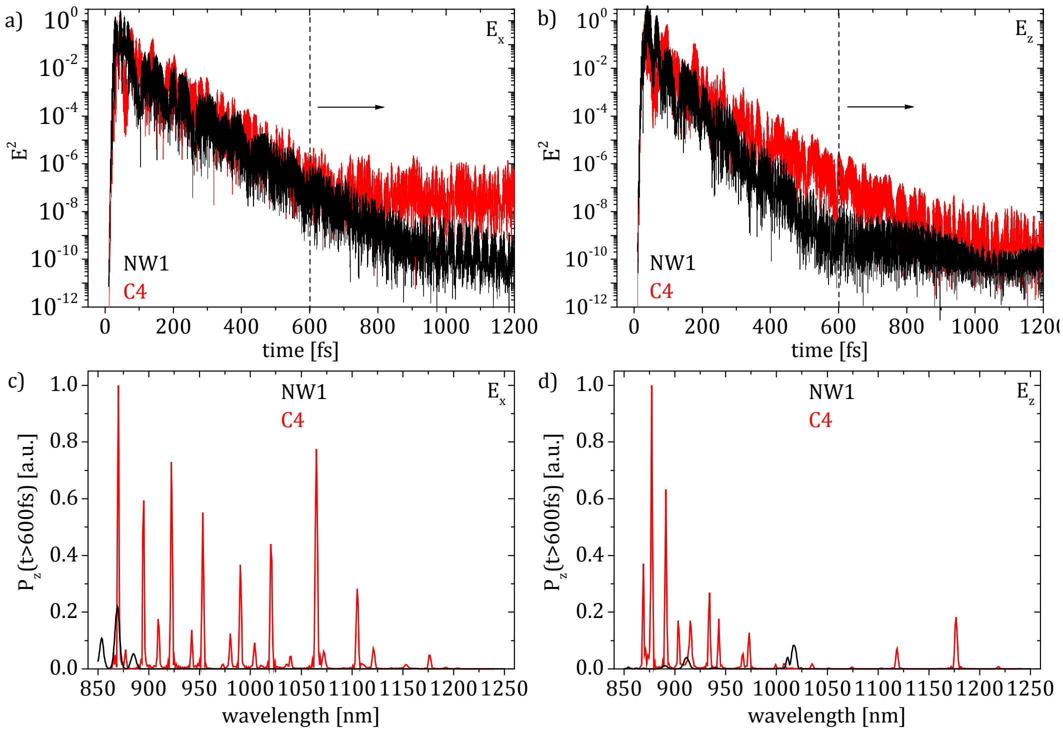

Supplementary information S4: The figure shows the good agreement between the peak positions in the experimental emission spectrum of C4 and the positions of radiative energy maxima P_z extracted from the numerical simulations. However, only about 63% of the peaks found experimentally are confirmed numerically. This can be explained by the fact that slight deviations in the geometry of the real SiNC C4 lead to the occurrence of additional modes and/or peak shifts that are not found by the simulations based on the ideal geometry of an inverted cone with dimensions as given in S1.

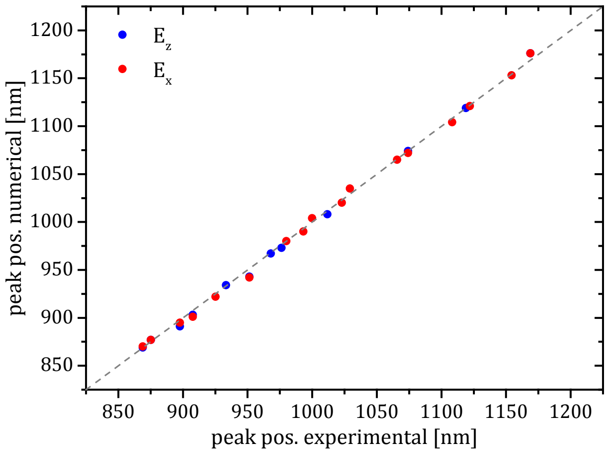

Supplementary information S5: Figure 3c in the manuscript displays the xy cross sectional energy density for the WGMs at 1153nm, 1119nm, 1176nm (2x), and 1104nm. The exemplary chosen modes typically represent the branches HE_{61}, HE_{81}, HE_{101}, HE_{81a}, and HE_{101a} identified in Fig. 3b. In HE_{ij}, the indices i and j correspond to the number of azimuthal and radial nodes, respectively[2]. With the additional index a, we distinguish the two appearance forms of HE_{81} and HE_{101}.

Supplementary information S6: The NA is estimated by a numerical analysis[3] of the far field radiation intensity of the modes in Fig. 3. We excite the SiNC C4 with a broadband dipole (E_x, as described in the main text) and monitor the radial distribution of the radiative energy in the z direction P_z at a distance of z_1=20nm and z_2=200nm above the top facet (see the right scheme below). The graph shows the normalized radial distribution of the radiative intensity from the 922nm mode for the two distances. For simplified analysis, we use the double distance of the outer maxima, i.e. 80nm, as the broadening b of the light cone between the distance of z_1=20nm and z_2=200nm away from the top facet. We apply

$$NA = n_{air} \cdot \sin(\tan^{-1}(b/\Delta z)) \qquad (5)$$

with $n_{air} \cong 1$ and $\Delta z = z_2 - z_1$ to estimate $NA = 0.22$.

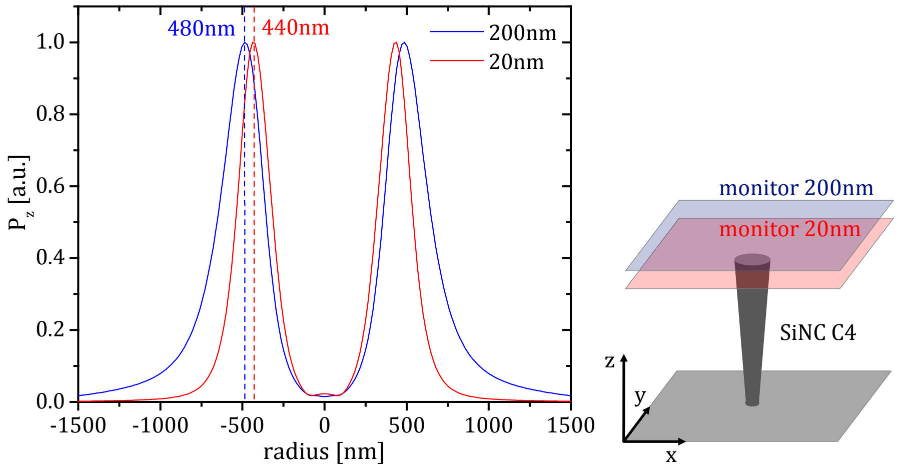

Supplementary information S7: The temporal change of carriers N in a unit volume of a solid state emitter is given by

$$\frac{dN}{dt} = G - R \qquad (6)$$

where G is the generation rate and R the sum of all carrier recombination processes. We rewrite G as

$$G = \frac{\eta_{abs} J}{eV} \qquad (7)$$

with η_{abs} representing the portion of the absorbed excitation intensity J, generating elementary charges e in the volume V of a SiNW or SiNC. R can be expressed as the sum of all radiative and non-radiative recombination processes

$$R = R_{sp} + R_{nr} + R_{st} \qquad (8)$$

where R_{sp} is the spontaneous (radiative) emission rate, R_{nr} is the sum of no non-radiative recombination processes (and carrier leakage) and R_{st} is the stimulated radiative emission. Assuming stationary conditions $dN/dt = 0$ and a low photon density leading to $R_{st} \cong 0$ the combination of (6), (7), and (8) leads to[4]

$$\frac{\eta_{abs} J}{eV} = R_{sp} + R_{nr} \qquad (9).$$

Using (9) and defining the radiative efficiency of the emitter as

$$\eta_{rad} = \frac{R_{sp}}{R_{sp} + R_{nr}} \qquad (10)$$

the internal spontaneous radiative emission power of light with wavelength λ, P_i

$$P_i = \frac{hc}{\lambda} V R_{sp} \qquad (11)$$

can be written as[4]

$$P_i = \eta_{rad} \eta_{abs} \frac{hc}{\lambda} J \qquad (12)$$

Here, hc/λ is the energy of a photon with wavelength λ and η_{abs} is absorbed fraction of the photon flux J injected by the pumping laser. To find the radiative emission P_i emitted in direction of the analyzer, an out-coupling efficiency η_{out} can be introduced, and accordingly[4]

$$P_e = \eta_{out} \eta_{rad} \eta_{abs} \frac{hc}{\lambda} J. \qquad (13)$$

Supplementary information S8: (10) can be rewritten as

$$\eta_{rad} = \frac{\tau_{sp}{}^{-1}}{\tau_{sp}{}^{-1} + \tau_{nr}{}^{-1}} \qquad (14)$$

in which τ_{nr} is the non-radiative recombination lifetime and τ_{sp} is the spontaneous emission lifetime. We calculate τ_{nr} for C4 an NW1 according to the approximation for SiNWs[5,6]

$$\frac{1}{\tau_{nr}} = \frac{1}{\tau_b} + \frac{4S}{d} \qquad (15)$$

where S is the surface recombination velocity, τ_b is the bulk lifetime and d the diameter of the SiNW. This approximation is valid for $S < \frac{2D}{d}$ where D is the diffusion constant of carriers in Si. Since $S = 5 \cdot 10^2 \frac{cm}{s}$ for an oxidized Si surface[7] and $\frac{2D}{d} \cong 7 \cdot 10^3 \frac{cm}{s}$ with $D = 36 \frac{cm^2}{s}$ and $d \cong 500nm$, using (8) is justified in the presented case. The bulk lifetime of carriers in crystalline Si (n-type, 1-5Ωcm) can range between $\tau_b \cong 10^{-4}s - 10^{-10}$[8,9]. For a similar bulk material quality it is strongly decreasing at high injection levels (Auger-effect), so the value of τ_{nr} in (8) for a low injection is determined by the (in this case) very low surface recombination lifetime $\tau_{nr} = 4S/d \cong 10^{-8}s$, where in contrast for a very high injection it will be dominated the bulk Auger-recombination. Since C4 and NW have similar surface properties (SiO_2-passivation) and bulk material quality and their volume and surface/volume ratio is roughly the same, we can expect their τ_{nr} to be comparable for the same injection conditions. These are in fact given for the compared experimental spectra (Fig. 2a,b) that both have been acquired under excitation with an 1.28mW CW laser at 660nm, for which both structures absorb about 40% of the light incident at the top facet (Fig. 1c).

τ_{sp} can be calculated as[10]

$$\tau_{sp} = \frac{1}{N \cdot B} \qquad (16)$$

where the radiative recombination probability $B = 1.1 \cdot 10^{-14} \frac{cm^3}{s}$ and N is the photo generated carrier density under optical pumping. Accordingly, τ_{sp} is dependent on intrinsic material properties and carrier injection, and therefore (as described above for τ_b) will be comparable for C4 and NW1 under the given experimental conditions. This means that if a further Purcell enhanced emission can be neglected, η_{rad} has about the same dimension for C4 and NW1.

Supplementary information S9: The mode volume of all modes determined in C4 (Fig. 3b) was estimated numerically using

$$V_m = \int (E^2 > \frac{E^2}{2}) dV \quad (17)$$

We find $0.01\mu m^3 < V_m < 0.05\mu m^3$ intuitively a higher mode volume for a higher orbit of the leaky WGMs in the structure. For the mode at λ=1027nm we find $V_m \cong 0.01\mu m^3$ (see below a visualization of the torus containing the ½ of the mode optical energy).

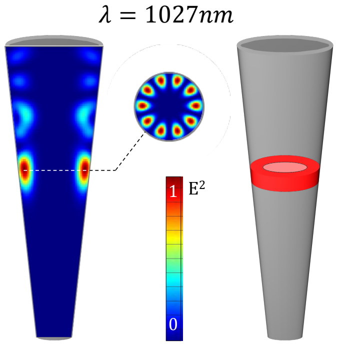

1. Olivero, J. & Longbothum, R. Empirical fits to the Voigt line width: A brief review. *J. Quant. Spectrosc. Radiat. Transf.* **17,** 233–236 (1977).

2. Li, B.-J. & Liu, P.-L. Numerical analysis of the whispering gallery modes by the finite-difference time-domain method. *IEEE J. Quantum Electron.* **32,** 1583–1587 (1996).

3. Lumerical Solutions Inc. (2014). at <https://www.lumerical.com/>

4. Coldren, N. A., Corzine, S. W. & Masanovic, M. L. *Diode lasers and photonic integrated circuits*. (John Wiley & Sons, 2012).

5. Dan, Y. *et al.* Dramatic reduction of surface recombination by in situ surface passivation of silicon nanowires. *Nano Lett.* **11,** 2527–32 (2011).

6. Allen, J. E. *et al.* High-resolution detection of Au catalyst atoms in Si nanowires. *Nat. Nanotechnol.* **3,** 168–173 (2008).

7. Stephens, W., Aberle, G. & Green, M. A. Surface recombination velocity measurements at the silicon–silicon dioxide interface by microwave-detected photoconductance decay. *J. Appl. Phys.* **76,** 363 (1994).

8. Tyagi, M. S. & Van Overstraeten, R. Minority carrier recombination in heavily-doped silicon. *Solid. State. Electron.* **26,** 577–597 (1983).

9. Richter, A., Glunz, S. W., Werner, F., Schmidt, J. & Cuevas, A. Improved quantitative description of Auger recombination in crystalline silicon. *Phys. Rev. B - Condens. Matter Mater. Phys.* **86,** 1–14 (2012).

10. Gerlach, W., Schlangenotto, H. & Maeder, H. On the radiative recombination rate in silicon. *Phys. status solidi* **13,** 277–283 (1972).
